# Trends in palliative care utilization among older adult decedents with and without cancer in Taiwan: a population-based comparative study

**DOI:** 10.1016/j.lanwpc.2025.101479

**Published:** 2025-01-28

**Authors:** Yu-Tai Lo, Tzu-Jung Chuang, Yu-Tung Huang, Yi-Lin Wu, Yi-Ching Yang, Chung-Yi Li

**Affiliations:** a Department of Geriatrics and Gerontology, National Cheng Kung University Hospital, College of Medicine, National Cheng Kung University, Tainan, Taiwan; b Department of Public Health, College of Medicine, National Cheng Kung University, Tainan, Taiwan; c Institute of Allied Health Sciences, College of Medicine, National Cheng Kung University, Taiwan; d National Centre for Geriatrics and Welfare Research, National Health Research Institute, Yunlin, Taiwan; e Department of Nursing, National Cheng Kung University Hospital, College of Medicine, National Cheng Kung University, Tainan, Taiwan; f Department of Family Medicine, School of Medicine, College of Medicine, National Cheng Kung University, Tainan, Taiwan

**Keywords:** Palliative care, Cause of death, Older adults, Patient acceptance of health care, Trends

## Abstract

**Background:**

The leading causes of death among the older adults have shifted from cancer to non-cancer conditions such as ischemia heart diseases, stroke, and dementia, affecting end-of-life care needs. This study examined the difference in the proportion of palliative care utilization in relation to specific causes of death and compared the trends in palliative care utilization between older adult decedents with and without cancer in Taiwan from 2010 to 2020.

**Methods:**

The study utilized data from the Health and Welfare Data Science Center in Taiwan, covering demographic and healthcare variables for 588,010 decedents aged 65+ years who died between 2010 and 2020. We used Poisson regression to investigate the temporal trends in palliative care utilization during the last six months of life. Multivariable logistic regression models were constructed to analyze cancer, and non-cancer causes of death associated with palliative care utilization.

**Findings:**

The proportion of palliative utilization in cancer deaths started at 21.7% in 2010 and increased to 63.2% by 2020 with the beta coefficient of 0.09 (95% CI: 0.09–0.09). The proportion of palliative utilization in non-cancer deaths started at 0.8% in 2010 and increased to 23.5% by 2020, with the beta coefficient of 0.26 (95% CI: 0.26–0.26). Compared to deceased cancer patients, deceased non-cancer individuals were less likely to have received palliative care (OR = 0.12, 95% CI: 0.12–0.13).

**Interpretation:**

Efforts to ensure equitable access to palliative care for non-cancer individuals should focus on expanding services, enhancing provider education, and promoting cultural sensitivity to meet the growing need for palliative care integration.

**Funding:**

This study was supported in part by grants from the 10.13039/501100004844National Cheng Kung University Hospital (NCKUH-11305001, NCKUH-V101-10) and the 10.13039/501100004737National Health Research Institutes (NHRI-13A1-CG-CO-04-2225-1). The sponsors had no influence on the study.

**Research in context**

**Evidence before this study**

We conducted a comprehensive review of the literature to identify studies examining palliative care utilization among older adults with cancer and non-cancer conditions. We searched multiple databases, including PubMed, MEDLINE, and Cochrane Library, for papers published in the period from January 2000 to December 2023, without language restrictions. Search terms included combinations of “palliative care,” “end-of-life care,” “cancer,” “non-cancer,” “older adults,” and “utilization trends.” Studies were included if they reported on palliative care utilization trends or factors influencing palliative care access among older adults. Conversely, studies were excluded if they focused solely on pediatric or non-terminal conditions. The evidence highlighted a significant increase in palliative care utilization among older adults with non-cancer conditions in Europe and North America, but there was a lack of comprehensive data from the West Pacific, where cultural, familial, and healthcare contexts differ significantly.

**Added value of this study**

This study provides the first detailed analysis of palliative care utilization trends among older adult decedents with cancer and non-cancer conditions in Taiwan over a decade (2010–2020). We found a substantial increase in palliative care utilization among individuals with cancer and those with non-cancer conditions, with a more pronounced annual growth rate among non-cancer decedents. Our findings reveal persistent disparities in palliative care access, with non-cancer decedents consistently less likely to have received palliative care compared to cancer decedents. This study underscores the urgent need for targeted strategies to address these disparities, including policy reforms and healthcare provider training to ensure equitable palliative care access for all older adults, irrespective of diagnosis.

**Implications of all the available evidence**

The findings from this study, combined with existing evidence, have significant implications for healthcare practice and policy in Taiwan and other aging societies. The growing proportion of older adults dying from non-cancer conditions necessitates the expansion of palliative care services beyond cancer care. Policymakers should prioritize integrating palliative care into routine management for all terminal conditions and focus on educating healthcare providers about the benefits of palliative care for non-cancer patients. Future research should explore the underlying causes of disparities in palliative care access and develop culturally sensitive interventions to promote equitable palliative care utilization across diverse patient populations. Our results could serve as a reference for other West Pacific countries facing similar demographic shifts and challenges in palliative care integration.

## Introduction

Advances in science, medicine, and public health have gradually changed the primary causes of death among older adults. Demographically, more than twice as many people die from non-cancerous causes than from cancer in European countries.^1^ The aging population faces increasing mortality from non-cancer conditions such as ischemic heart disease, stroke, Alzheimer's and other dementias, and chronic obstructive pulmonary diseases,^2^ which require comprehensive end-of-life (EOL) care strategies. The dramatic shift in the leading causes of death affects the proportion of patients cared for by palliative care and the patterns of utilization. While palliative care has traditionally focused on cancer patients,^3^ the needs of individuals with non-cancer terminal illnesses are becoming more urgent,^4^^,^^5^ especially as Taiwan approaches super-aged society status by 2025, when over 20% of the population will be aged 65 or older.^6^ Although cancer remains the leading cause of death in Taiwan, the number of older adults dying from heart disease, pneumonia, and cerebrovascular disease are increasing.^7^ This demographic shift raises pressing questions about equitable access to high-quality EOL care for older adults with diverse health conditions.

Evidence shows that palliative care reduces intensive medical interventions and alleviates symptoms for EOL patients with both cancer and non-cancer illnesses.^8^ However, disparities in palliative care utilization persist between these groups, particularly among oldest age groups.^9^ Cagle et al. reported that cancer patients are five times more likely to receive palliative care than those with non-cancer illnesses.^3^ As awareness grows, providers and researchers have been working to develop strategies that improve access to palliative care for older adults with non-cancer diagnoses. Barriers include stigma and misconceptions about palliative care, lack of clear referral guidelines, healthcare system limitations, financial, and social obstacles.^4^^,^^9^ In many Asian countries, palliative care services focus on cancer patients due to limited resources and prioritization.^10^ Therefore, additional information on how specific diagnoses impact palliative care utilization is essential for improving access for non-cancer patients and strengthening support systems.

Previous studies have primarily relied on data from Europe and North America.^3^^,^^4^^,^^8^^,^^9^^,^^11^ In the United States, patients with heart failure or dementia represent the fastest-growing group of hospice users, with the proportion of non-cancer hospice patients increasing from 34.6% in 1995 to 69.9% in 2017.^3^ A German study reported that between 2014 and 2016, 39.4% of non-cancer patients and 85.6% of cancer patients received palliative care in their last year of life.^5^ However, little is known about EOL palliative care utilization trends among decedents with cancer and non-cancer conditions in Asia, where cultural/familial, and religious contexts differ significantly from those in Western societies. Moreover, few studies have examined palliative care distribution across specific non-cancer conditions.^3^^,^^12^ Understanding palliative care utilization trends is essential for healthcare system structure, service delivery, and reimbursement policies, which can vary widely between countries. To support the development of innovative, collaborative services for high-quality EOL care worldwide, reliable population-based estimates across countries are required to capture the systematic variations in palliative care utilization over time among older adults with different life-threatening conditions.

In recent decades, Taiwan has expanded its palliative care services under the National Health Insurance (NHI) system, with services initiated in home care settings in 1996, followed by the introduction of inpatient palliative care units in 2000, and shared-care services in 2011.^13^ Initially focused on terminal cancer care, palliative services in Taiwan have included eight additional terminal non-cancer illnesses, such as dementia, heart failure, and chronic obstructive lung disease, since 2009. Until now, few studies have examined trends in palliative care utilization between cancer and non-cancer illnesses in Taiwan. To address this knowledge gap, this study aimed to investigate the difference in the proportion of palliative care utilization in relation to specific causes of death and compare the trends in palliative care utilization between older adults with cancer and those without cancer who died between 2010 and 2020 in Taiwan. We also assessed the potential effect-modifications by each of the selected covariates on the relationship between cancer/non-cancer status and palliative care utilization. The findings may offer insights to guide policy developments and improve palliative care equity for aging populations in Taiwan and other Asian countries.

## Methods

### Study design and data sources

We conducted an observational study using Taiwan’s NHI claims data supervised by the Health and Welfare Data Science Center (HWDSC) and the Multiple Causes of Death Registry between 2009 and 2020. Taiwan’s NHI is a single-payer system; since official implementation in 1995, its coverage rate has reached over 99% of the population.^14^ The NHI claims contain personal medical and extensive healthcare information of 23 million individuals, including primary demographic characteristics, original clinical records, inpatient time, surgery, treatment/medication, medical expenditure, and diagnostic codes based on the International Classification of Disease, 9th and 10th Revisions, Clinical Modification (ICD-9-CM/ICD-10-CM).^13^ NHI claims provide details of a comprehensive and long-term follow-up period of claim data for each beneficiary. All personal information was anonymized and de-identified before access. Data on the date and cause of death were collected from the Multiple Causes of Death database. In Taiwan, it is a legal requirement to register all live births and deaths within 10 days following the event. Death certificates provide a range of information, including the underlying cause of death and various demographic variables. The Multiple Causes of Death database has been evaluated for data quality and deemed valid.^15^^,^^16^ NHI claims, and the Multiple Causes of Death Registry were linked using encrypted patient identifiers. The data were collected from August 2023 to June 2024. This study was conducted according to the STROBE guidelines (Supplementary Table S1).

### Study population

This study adopted an unselected study sample. The study population comprised 1,796,997 individuals who died between January 1, 2010, and December 31, 2020, in Taiwan. The NHI assigns related diagnostic codes for palliative care based on the ICD-9-CM/ICD-10-CM for non-cancerous terminal conditions (Supplementary Table S2). The inclusion criteria were as follows: (1) age ≥ 65 years at death and (2) an underlying cause of death on the death certificate compatible with indications for palliative care services (Supplementary Table S2). We included all decedents regardless of their place of death. The exclusion criteria were as follows: (1) individuals without records of inpatient or emergency utilization within six months before death (n = 39,268); (2) missing demographic data such as sex, region of residence, and insurance premium (n = 2215); or (3) missing data on hospital-level characteristics (n = 11,685). We excluded decedents without records of inpatient or emergency utilization within the last six months of life because it may be unreasonable to classify them as being in a terminal state requiring palliative care under the NHI system (e.g., sudden death). A flowchart illustrating the participant selection process is presented in Supplementary Fig. S1.

### Ethics statement

The ethics committee of the National Cheng-Kung University approved this study (B-ER-111-214). The requirement for patient consent was waived because the identity of all the patients in the HWDSC was concealed. All procedures were performed in accordance with the Declaration of Helsinki and its later amendments.

### Explanatory variables

The underlying causes of death were identified using the corresponding ICD-9/ICD-10 codes from the death certificates. We considered cancer and terminal non-cancer conditions that qualified for palliative care under the NHI regulations.^17^ We further grouped chronic obstructive lung diseases and other terminal lung diseases into “lung diseases,” chronic kidney disease and acute renal failure into “renal diseases,” and various terminal motor neuron diseases into “others” due to the small number of cases in those categories.

### Outcome variable

The primary outcome, palliative care utilization (yes/no), was based on the payment procedure codes extracted from the NHI claims within the last six months of life. We considered all three types of palliative care services, namely, inpatient care units, shared care, and home care, as palliative care utilization. Decedents with records of any of the three payment procedure codes were considered palliative care users.

### Covariates

Information on demographic and socioeconomic status-related factors, including age, sex, and salary-based insurance premiums, and the level of urbanization, was obtained from the beneficiary records of the NHI claims. The deceased individuals were categorized into six age groups: 65–69, 70–74, 75–79, 70–84, 85–90, and ≥90 years. Aging is highly heterogeneous, and significant differences in health status, functional abilities, and disease risk emerge over even short time spans among older adults. Grouping older populations in five-year intervals allows researchers to capture these nuances more precisely, especially in studies involving health status and care needs.^18^ Additionally, many large-scale epidemiological studies and health surveys use five-year age intervals for older adults. This convention enables direct comparability of findings across studies and aligns with widely used datasets, making it easier to analyze and interpret data in the context of global aging research.^19^ We categorized salary-based insurance premiums into two levels based on the median of insurance premiums: less than 25,000 New Taiwan dollars (NTD) and greater than 25,000 NTD. The urbanization level in the region of residence was categorized into three levels based on the classification scheme proposed by Liu et al.,^20^ which considered factors such as population density, proportion of college-educated residents, percentage of older adults, proportion of the agricultural workforce, and the number of physicians per 105 residents. The hospital levels also vary in their policies and treatment recommendations and were shown to associate with palliative care utilization in Taiwan.^21^ Moreover, the distribution of hospital levels has changed over time.^21^ We initially categorized hospital levels according to accreditation level, namely, medical centers, regional hospitals, district hospitals, and home healthcare institutions. However, only 45 decedents received palliative care from home healthcare institutions during the study period. Therefore, we combined home healthcare institutions with district hospitals for analysis.

### Statistical methods

Descriptive statistics (frequencies/percentages, means/standard deviations [SD]) were used to summarize the full sample and the proportion of palliative care utilization among older decedents according to significant characteristics. The annual overall and specific proportions of palliative care utilization were estimated by dividing the number of deceased individuals who had received palliative care by the total number of deceased individuals in the NHI program from 2010 to 2020. We calculated overall crude palliative care utilization and the 95% confidence interval (CI) using Agresti-Coull method.^22^ We further adopted direct standardization, using the WHO 2000 standard population, to calculate age-sex-standardized proportion of palliative care utilization over time.

Poisson regression models were used to investigate whether temporal trends in palliative care utilization existed over the study period. We constructed Poisson regression models using log function as the link function, with the proportion of palliative care utilization as the response variable. In the analysis of secular trends in palliative care utilization, the year of death was used as an independent variable. The model was fitted separately for patients with cancer and non-cancer, and the interaction between cause of death and year of death was further assessed. In the analysis of the association between cause of death and palliative care utilization, causes of death (i.e., cancer/non-cancer status or seven non-cancer conditions: dementia, stroke, heart failure, liver diseases, lung diseases, renal diseases, and others) were used as independent variables. This analysis was further stratified according to selected covariates. The linearity assumption underlying the Poisson regression model for quantitative predictors was assessed by adopting the visual inspection of predictor vs. response method. A scatter plot of the trend against the log-transformed proportion of palliative care utilization revealed that a log-linear trend was reasonable (Supplementary Fig. S2).

Simple logistic regression models were used to assess the unadjusted association between the selected causes of death and palliative care utilization. We further constructed multivariable logistic regression models to estimate the odds ratios (ORs) and 95% confidence intervals (CIs) of palliative care utilization before death in association with cause of death and selected covariates. We identified confounders according to previous literature and drew a causal directed acyclic graph based on these confounders (Supplementary Fig. S3). The multivariable logistic regression models were adjusted for age at death, sex, year of death, salary-based insurance premium, urbanization level, and hospital level. We also performed logistic regression analyses to assess the interaction between cancer status and palliative care utilization according to various selected covariates. Interaction terms between cancer status and each covariate were included in the regression model, and significance testing was conducted for the regression coefficients of the interaction terms. All statistical analyses were performed using the SAS software (SAS Institute, Cary, NC, USA). Statistical significance was determined at a two-tailed p < 0.05.

### Role of the funding source

The National Cheng Kung University Hospital and the National Health Research Institute provided funding for the study and had no role in the study design, data collection, analysis, interpretation, or writing of the manuscript.

## Results

### Sample description

Overall, 588,010 decedents between 2010 and 2020 were included with an average age of 80.6 years (SD = 8.3 years) at death, and most were male (58.0%; Table 1). Half of the deaths (50.3%) were cancer-related. Among them, 175,257 were palliative care users with an average age of 78.8 years (SD = 8.3 years) at death with 82.3% of the deaths among palliative care users being cancer-related.

**Table 1:** Palliative care utilization status of older adults who died between 2010 and 2020 according to characteristics.

	Total sample (N = 588,010)		Palliative care users (N = 175,257)		Proportion of palliative care use	(95% CI)%
	n/mean	%/SD	n/mean	%/SD		
**Characteristics**						
Age (years)	80.6	8.3	78.8	8.3	–	–
65–69	78,293	13.3	32,226	18.4	41.2%	40.8%–41.5%
70–74	85,939	14.6	30,385	17.3	35.4%	35.0%–35.7%
75–79	108,920	18.5	34,508	19.7	31.7%	31.4%–32.0%
80–84	124,832	21.2	33,962	19.4	27.2%	27.0%–27.5%
85–89	109,969	18.7	26,673	15.2	24.3%	24.0%–24.5%
90+	80,057	13.6	17,503	10.0	21.9%	21.6%–22.2%
Sex						
Male	340,944	58.0	99,531	56.8	29.2%	29.0%–29.3%
Female	247,066	42.0	75,726	43.2	30.7%	30.5%–30.8%
Cause of death						
Cancer	295,754	50.3	144,280	82.3	48.8%	48.6%–49.0%
Non cancer	292,256	49.7	30,977	17.7	10.6%	10.5%–10.7%
Heart disease	44,872	7.6	3972	2.3	8.9%	8.6%–9.1%
Dementia	13,704	2.3	2514	1.4	18.3%	17.7%–19.0%
Lung diseases	60,047	10.2	5230	3.0	8.7%	8.5%–8.9%
Stroke	98,622	16.8	9329	5.3	9.5%	9.3%–9.6%
Kidney diseases	38,598	6.6	5277	3.0	13.7%	13.3%–14.0%
Liver diseases	22,587	3.8	3666	2.1	16.2%	15.8%–16.7%
Others	13,826	2.4	989	0.6	7.2%	6.7%–7.6%
Insurance premium (NTD)						
≤25,000	455,807	77.5	131,875	75.2	28.9%	28.8%–29.1%
>25,000	132,203	22.5	43,382	24.8	32.8%	32.6%–33.1%
Level of urbanization						
High	185,616	31.6	61,199	34.9	33.0%	32.8%–33.2%
Medium	173,007	29.4	50,736	29.0	29.3%	29.1%–29.5%
Low	229,387	39.0	63,322	36.1	27.6%	27.4%–27.8%
Hospital level						
Medical center	194,829	33.1	80,033	45.7	41.1%	40.9%–41.3%
Regional hospital	277,277	47.2	81,952	46.8	29.6%	29.4%–29.7%
District hospital	115,904	19.7	13,272	7.6	11.5%	11.3%–11.6%

NTD: New Taiwan Dollars; 1 USD = 32 NTD.

### Proportion of palliative care utilization

The overall proportion of palliative care utilization was 29.8%; the utilization proportion decreased as the age at death increased (Table 1). The crude proportion of palliative care utilization varied across different demographics and disease categories. Younger age groups had a higher proportion than older age groups. The highest proportion of palliative care utilization was 41.2% in the 65–69-year age group, while the lowest was 21.9% in the 90+ age group. A slightly higher proportion was noted among females (30.7%) compared to males (29.2%). Deceased cancer patients had a higher proportion (48.8%) compared to non-cancer deceased individuals (10.6%). Among non-cancer causes of death, dementia (18.3%), liver disease (16.2%), and kidney disease (13.7%) were associated with higher proportions of palliative care utilization than stroke (9.5%), heart disease (8.9%), lung disease (8.7%), and others (7.2%).

### Trends of palliative care utilization

The crude proportion increased from 11.4% in 2010 to 44.3% in 2020. The age- and sex-standardized proportion increased from 13.1% in 2010 to 48.9% in 2020. Both the crude and standardized proportions showed a steady upward trajectory annually (Table 2).

**Table 2:** Secular trends in the proportion of palliative care utilization among older adults in Taiwan, 2010–2020.

Variables	Proportion by calendar year, %										
	2010	2011	2012	2013	2014	2015	2016	2017	2018	2019	2020
Crude proportion	11.4 (11.2–11.7)	14.5 (14.2–14.8)	19.3 (18.9–19.6)	22.5 (22.1–22.9)	25.3 (25.0–25.7)	30.4 (30.0–30.8)	33.9 (33.5–34.3)	37.2 (36.8–37.6)	39.1 (38.7–39.5)	41.4 (41.0–41.8)	44.3 (43.9–44.7)
Standardized proportion^a^	13.1 (12.6–13.5)	16.9 (16.4–17.3)	22.7 (22.1–23.2)	26.6 (26.0–27.2)	30.0 (29.4–30.6)	35.4 (34.8–36.1)	39.5 (38.8–40.1)	42.5 (41.8–43.2)	44.0 (43.3–44.7)	46.3 (45.6–47.0)	48.9 (48.2–49.6)

a Standardized proportion adjusted for age and sex.

**Fig. 1:**
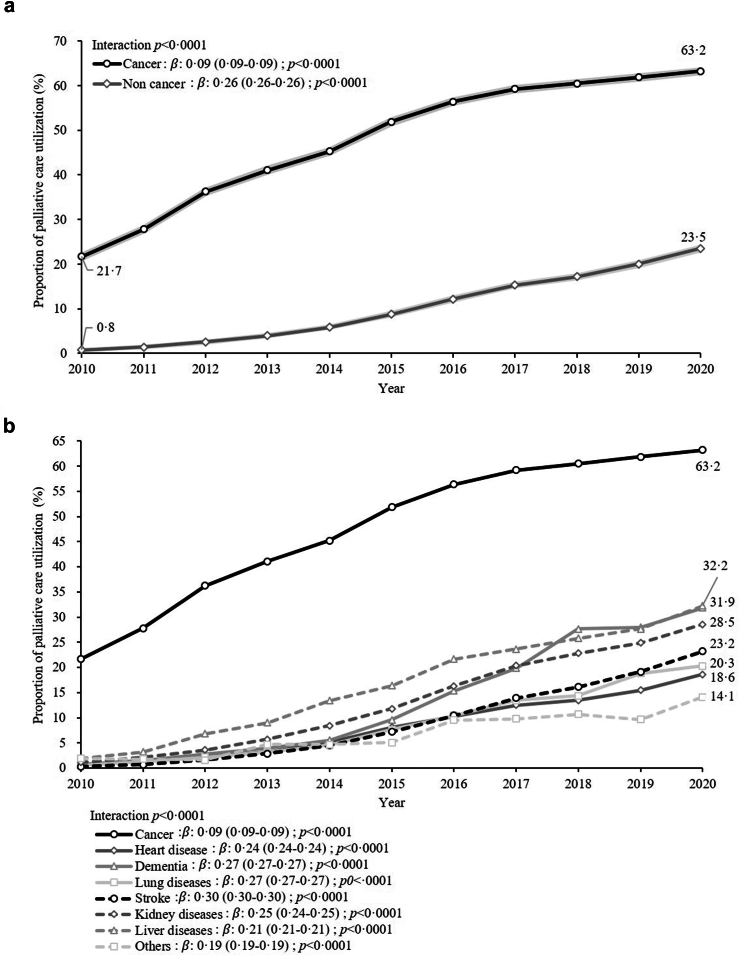
Trends of proportion of palliative care utilization among older decedents between 2010 and 2020. (a) Proportion of palliative care utilization for cancer and noncancer-related deaths (shaded area refers to 95% confidence band). (b) Proportion of palliative care utilization according to various causes of deaths.

Palliative care utilization increased for both cancer- and non-cancer-related deaths between 2010 and 2020 (Fig. 1a). Individuals diagnosed with cancer consistently utilized palliative care at higher rates compared to those without cancer. The proportion of palliative care utilization in cancer deaths was 21.7% in 2010 and increased steadily each year, reaching 63.2% in 2020. The proportion of palliative utilization in non-cancer deaths started at 0.8% in 2010 and rose to 23.5% in 2020. The interaction between cancer status and year of death in the Poisson regression models was statistically significant (p < 0.0001), with non-cancer deaths having a larger beta coefficient (0.26) than cancer deaths (0.09), indicating a significant difference in the trends of the proportion of palliative care utilization between the two groups. Palliative care utilization for various non-cancer causes of death also showed an upward trend from 2010 to 2020; however, the beta coefficient (0.19–0.30) varied among the non-cancer diagnoses (Fig. 1b). The differences in the trends among the various non-cancer causes of death were also statistically significant (p < 0.0001).

### Factors associated with palliative care utilization

Compared to individuals who died from cancer, people who died from non-cancer causes were less likely to receive palliative care before death (OR = 0.12, 95% CI: 0.12–0.13). In the fully adjusted Model 2, compared to cancer death, non-cancer deaths including dementia (OR = 0.20, 95% CI: 0.19–0.21), liver diseases (OR = 0.19, 95% CI: 0.19–0.20), kidney diseases (OR = 0.16, 95% CI: 0.15–0.16), lung diseases (OR = 0.12, 95% CI: 0.11–0.12), stroke (OR = 0.11, 95% CI: 0.10–0.11), heart diseases (OR = 0.10, 95% CI: 0.09–0.10), and others (OR = 0.08, 95% CI: 0.07–0.08) were all associated with significantly lower odds of receiving palliative care (Table 3). Regardless of the treatment of age and calendar year as either continuous or categorical variables, the logistic regression models produced consistent results (Supplementary Table S3).

**Table 3:** Factors associated with palliative care utilization.

Factors	Unadjusted OR (95% CI)	p value	Adjusted OR Model 1 (95% CI)	p value	Adjusted OR Model 2 (95% CI)	p value
**Cause of death (Reference: cancer)**						
Non-cancer	0.12 (0.12–0.13)	<0.0001	0.12 (0.12–0.13)	<0.0001	–	
Heart disease	0.10 (0.10–0.11)	<0.0001	–		0.10 (0.09–0.10)	<0.0001
Dementia	0.24 (0.23–0.25)	<0.0001	–		0.20 (0.19–0.21)	<0.0001
Lung diseases	0.10 (0.10–0.10)	<0.0001	–		0.12 (0.11–0.12)	<0.0001
Stroke	0.11 (0.11–0.11)	<0.0001	–		0.11 (0.10–0.11)	<0.0001
Kidney diseases	0.17 (0.16–0.17)	<0.0001	–		0.16 (0.15–0.16)	<0.0001
Liver diseases	0.20 (0.20–0.21)	<0.0001	–		0.19 (0.19–0.20)	<0.0001
Others	0.08 (0.08–0.09)	<0.0001	–		0.08 (0.07–0.08)	<0.0001
**Age group (years) (Reference: 65–69)**						
70–74	0.78 (0.77–0.80)	<0.0001	0.96 (0.94–0.99)	0.0016	0.97 (0.95–0.99)	0.0043
75–79	0.66 (0.65–0.68)	<0.0001	0.91 (0.89–0.93)	<0.0001	0.92 (0.90–0.94)	<0.0001
80–84	0.53 (0.52–0.54)	<0.0001	0.88 (0.86–0.90)	<0.0001	0.90 (0.88–0.92)	<0.0001
85–89	0.46 (0.45–0.47)	<0.0001	0.86 (0.84–0.88)	<0.0001	0.88 (0.86–0.90)	<0.0001
90+	0.40 (0.39–0.41)	<0.0001	0.90 (0.88–0.93)	<0.0001	0.93 (0.91–0.96)	<0.0001
**Sex (Reference: Male)**						
Female	1.07 (1.06–1.08)	<0.0001	1.21 (1.19–1.22)	<0.0001	1.20 (1.18–1.22)	<0.0001
**Year of death (Reference: 2010)**						
2011	1.31 (1.27–1.37)	<0.0001	1.39 (1.34–1.45)	<0.0001	1.39 (1.34–1.45)	<0.0001
2012	1.85 (1.78–1.92)	<0.0001	2.06 (1.98–2.14)	<0.0001	2.06 (1.98–2.14)	<0.0001
2013	2.25 (2.17–2.33)	<0.0001	2.58 (2.49–2.68)	<0.0001	2.58 (2.49–2.68)	<0.0001
2014	2.62 (2.53–2.72)	<0.0001	3.16 (3.05–3.28)	<0.0001	3.16 (3.04–3.28)	<0.0001
2015	3.38 (3.27–3.50)	<0.0001	4.28 (4.12–4.44)	<0.0001	4.28 (4.12–4.44)	<0.0001
2016	3.97 (3.84–4.11)	<0.0001	5.40 (5.21–5.60)	<0.0001	5.40 (5.21–5.60)	<0.0001
2017	4.59 (4.44–4.75)	<0.0001	6.43 (6.20–6.66)	<0.0001	6.42 (6.20–6.66)	<0.0001
2018	4.97 (4.81–5.14)	<0.0001	7.06 (6.81–7.32)	<0.0001	7.08 (6.82–7.34)	<0.0001
2019	5.46 (5.28–5.64)	<0.0001	7.98 (7.70–8.27)	<0.0001	7.97 (7.68–8.26)	<0.0001
2020	6.15 (5.95–6.36)	<0.0001	9.10 (8.78–9.43)	<0.0001	9.07 (8.75–9.41)	<0.0001
**Insurance premium (NTD) (Reference: ≤25,000)**						
>25,000	1.20 (1.18–1.22)	<0.0001	1.01 (0.99–1.02)	0.3313	1.01 (0.99–1.02)	0.3163
**Level of urbanization (Reference: Low)**						
Medium	1.09 (1.07–1.10)	<0.0001	0.90 (0.88–0.91)	<0.0001	0.90 (0.88–0.91)	<0.0001
High	1.29 (1.27–1.31)	<0.0001	1.02 (1.00–1.03)	0.0367	1.02 (1.00–1.03)	0.0699
**Hospital level (Reference: Medical center)**						
Regional hospital	0.60 (0.59–0.61)	<0.0001	0.71 (0.70–0.72)	<0.0001	0.71 (0.70–0.72)	<0.0001
District hospital	0.19 (0.18–0.19)	<0.0001	0.26 (0.25–0.26)	<0.0001	0.26 (0.25–0.26)	<0.0001

NTD: New Taiwan Dollars; 1 USD = 32 NTD.

**Fig. 2:**
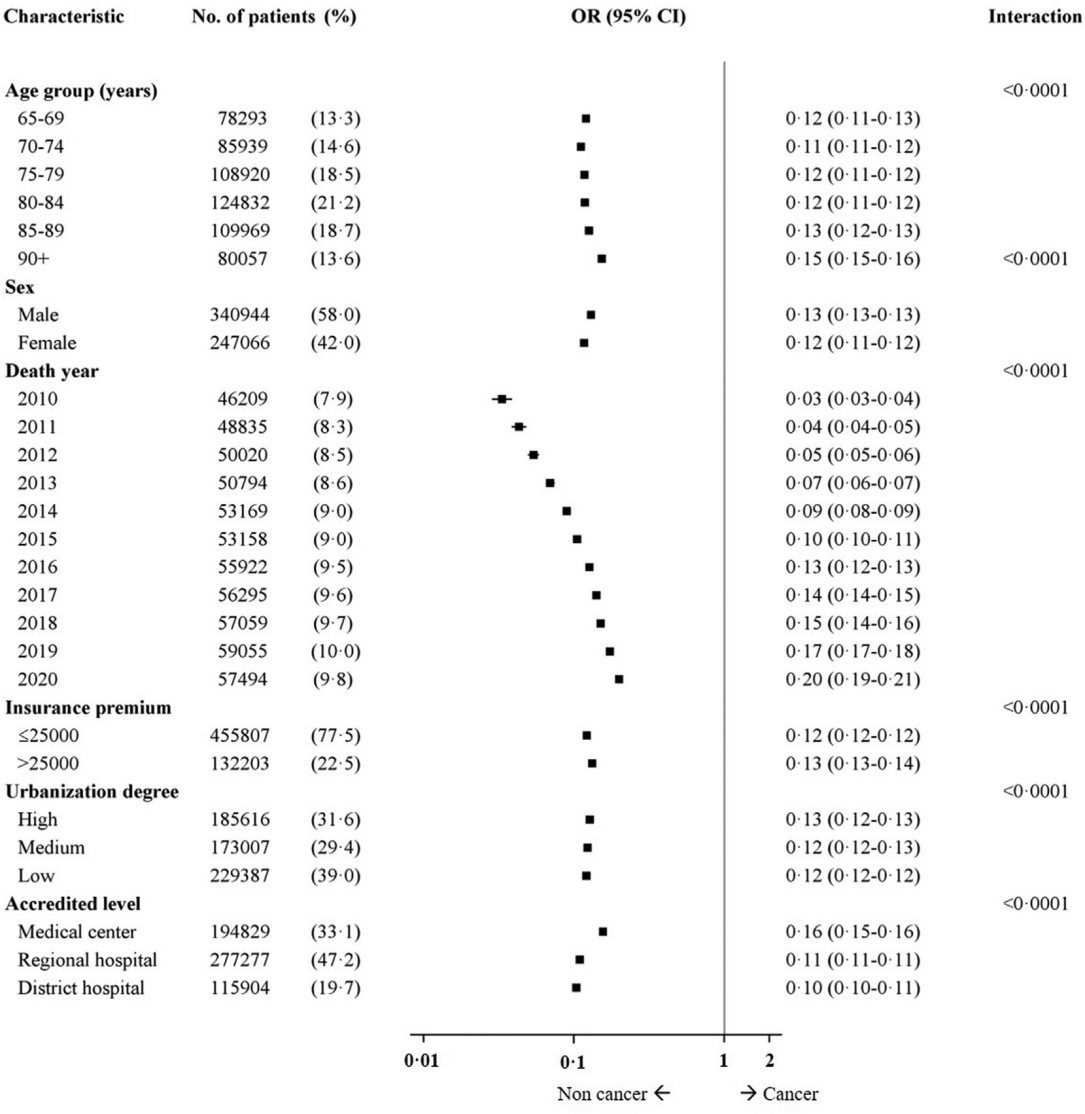
The interactions of the cause of death and palliative care utilization within subgroups.

Compared with cancer decedents, non-cancer decedents were consistently less likely to receive palliative care among different subgroups according to age, sex, year of death, insurance premium, degree of urbanization, and accredited level of medical facilities (Fig. 2). The interactions of the cause of death with the above covariates were all statistically significant.

## Discussion

The findings of this study highlight significant increases in the proportion of palliative care utilization in older adults with both cancer and non-cancer causes of death in Taiwan from 2010 to 2020. To the best of our knowledge, this study is the first to examine the trends of palliative care utilization among deceased individuals with cancer or various non-cancer conditions in Asia. The results revealed a consistent and substantial increase in the proportion of palliative care utilization, with notable growth in the proportion of decedents with non-cancer causes of death. Despite the overall increased proportion of palliative care utilization, significant disparities persist across age, sex, insurance premium, urbanization level, and hospital-level subgroups. This is particularly pronounced for deceased individuals with non-cancer illnesses, as they were less likely to have received palliative care compared to deceased cancer patients. These findings underscore the need for improvements in palliative care access and delivery, particularly for deceased individuals with non-cancer illnesses.

Palliative care, acknowledged as part of the human right to health,^23^ has developed unevenly globally.^24^ While approximately 58% of countries have at least one palliative care service, significant regional disparities persist.^24^ Demand for palliative care is anticipated to rise by 87% by 2060.^25^ In the United States, utilization has been growing among hospitalized patients with chronic, non-cancer conditions, with 52.8% of older adults now receiving hospice care.^3^^,^^26^ Similarly, the Canadian Institute for Health Information reported that 58% of decedents received some form of palliative care between 2021 and 2022.^27^

In Taiwan, our study shows a significant increase in palliative care utilization, from 11.4% in 2010 to 44.3% in 2020. Despite this progress, palliative care remains underutilized compared to Western countries. This increase reflects growing acceptance and integration of palliative care services over the past decade, aided by Taiwan’s NHI policy expansions since 2009. These policies have broadened coverage to include a range of terminal illnesses and established three distinct palliative care models, advancing access beyond cancer patients. In 2013, amendments to the Hospice Palliative Care Act allowed the withdrawal of artificial ventilation for terminally ill patients, positioning Taiwan as the first country in Asia to legalize life-sustaining treatment withdrawal at EOL.^28^^,^^29^ The Patient Right to Autonomy Act, enacted in 2019, further strengthened patient autonomy, promoting a supportive environment for palliative care.^30^ Additionally, NHI reimbursement for community-based palliative care introduced in 2014 has improved accessibility and reduced costs.^31^ Our findings emphasize the importance of continued investment in palliative care policy and capacity building to sustain positive trends.

However, disparities remain. In our study, palliative care utilization among non-cancer deaths was relatively low—18.4% for dementia, 8.7% for lung diseases, and 8.9% for heart diseases—compared to higher rates in Western countries, where palliative care utilization exceeds 20% for COPD and heart disease and 40% for dementia in the UK.^4^ Variation across countries in palliative care utilization for cancer and non-cancer patients is influenced by factors such as access, awareness, and healthcare policy frameworks. For example, in Germany, the proportion of non-cancer patients receiving palliative care rose from 3.5% to 8.1% between 2007 and 2011.^32^ In underdeveloped and developing countries in Africa, Asia, and Latin America, palliative care for cancer patients often addresses service gaps left by limited oncological resources, and non-cancer patients face restricted access due to limited resources and trained personnel.^33^

Moreover, palliative care utilization for non-cancer decedents in Taiwan appears to lag approximately a decade behind that of cancer patients. For instance, in 2020, palliative care utilization for non-cancer patients (23.5%) was roughly equivalent to the rate observed among cancer patients in 2010 (21.7%). These disparities underscore the ongoing challenges in achieving equitable access to palliative care, even with universal health insurance coverage for palliative services in Taiwan. Issues such as geographical disparities, workforce shortages, and misunderstandings about palliative care among both patients and healthcare providers persist.^9^ In rural Taiwan, community nurses are often willing to provide palliative care but tend to limit their services to consultation and referral due to training constraints and a lack of resources for in–home visits and bereavement support.^34^ Consequently, educational programs to enhance knowledge and practical skills in palliative care are essential for these healthcare providers. Additionally, research indicates challenges in sustaining long-term dedication among palliative care physicians in Taiwan, as many providers have short practice periods, with only a minority maintaining continuous involvement.^35^ Efforts to improve physician retention in palliative care are ongoing to ensure quality care, especially given the significant demand due to an aging population. Targeted policies could help address these issues by increasing awareness of palliative care’s benefits and potentially adjusting Taiwan’s NHI support systems to promote the integration of palliative care into primary care settings. Future research should explore specific barriers to palliative care access, including lack of awareness and cultural biases, and evaluate interventions that could improve utilization rates. These efforts would support equitable access to EOL care services for all terminally ill patients, aligning with the NHI’s objective to provide comprehensive care.

The cause and year of death were among the strongest predictors of the proportion of palliative care utilization in the fully adjusted model. We found that deceased non-cancer patients were 88% less likely to have utilized palliative care compared to deceased cancer patients, which is consistent with previous studies.^3^^,^^4^^,^^9^^,^^11^ In considering the interaction effects within our logistic model, it is important to recognize that while multiplicative interactions—such as those evaluated by odds ratios—are common in logistic regression frameworks, additive interaction is particularly meaningful for public health implications. Additive interaction assesses the absolute risk differences that could guide targeted interventions.^36^ This approach aligns with recommendations that prioritize additive over multiplicative interaction to provide insights into public health measures that could reduce disparities in palliative care access for both cancer and non-cancer patients. Future studies should consider this perspective to improve the practical applicability of palliative care strategies across diverse patient populations.

The low utilization of palliative care services by patients with non-cancer illnesses from clinical perspectives may stem from challenges in accurately predicting the prognosis of these conditions (e.g., heart failure, dementia).^37^ The disease course tends to be more predictable for cancer than for non-cancerous diseases, making it easier for physicians to estimate the life expectancy of cancer patients who meet the criteria for the initiation of palliative care. Additionally, healthcare providers for many common non-cancer illnesses may not fully understand the benefits of palliative care services and, hence, may not recommend these services to patients who could benefit from them.^38^ These findings imply that improving the education and training of healthcare providers regarding palliative care benefits and their applicability to non-cancer conditions is essential. Furthermore, healthcare systems should develop guidelines that facilitate earlier identification and referral for palliative care among patients with non-cancer diseases, potentially through multidisciplinary collaboration.

Our findings indicate that patients who died from dementia were more likely to receive palliative care before death compared to those who died from other non-cancer causes. Supporting this, Luth et al. found that dementia patients in the United States had 45% higher odds of receiving timely hospice services.^39^ Patients with dementia have higher odds of receiving palliative care for several reasons reflecting the unique needs and challenges associated with their condition. Patients with dementia often experience a range of symptoms, including pain, anxiety, and difficulty in swallowing, which require palliative care to improve their quality of life.^40^^,^^41^ Additionally, dementia’s progressive and incurable nature, combined with associated functional decline and the high burden on caregivers, likely heightens awareness among clinicians, patients, and families regarding the benefits of palliative care.^42^ These findings imply that healthcare systems should continue to integrate palliative care into dementia care pathways to ensure that patients with dementia have timely access to these services. By addressing the specific needs of dementia patients and their caregivers through palliative care, healthcare providers can enhance the quality of life for these individuals and support their families throughout the disease trajectory.

Our finding that deceased older adults were less likely to have utilized palliative care services before death differs from the findings of two recent studies.^3^^,^^43^ Cagel et al. found that older adults were more likely to receive hospice services before death in a population-based sample in the United States, irrespective of the type of disease (cancer/non-cancer).^3^ Buck et al. reported that the majority of patients accessing a hospice at-home service in the UK were aged 78 years or older.^43^ The discrepant effect of age on palliative care utilization may be related to cultural variations. Research indicates that many Chinese older adults prefer to delegate EOL care decisions to their families, reflecting cultural values that prioritize family involvement and harmony. Similarly, Ke et al. found that nearly 60% of Taiwanese older adults preferred their spouses, adult children, or healthcare professionals to make their EOL decisions.^44^ The value of filial piety affects help-seeking behavior, caregiving, family relationships, and caregiver health in Chinese families.^45^ Therefore, older age was found to be a barrier to palliative care utilization in Taiwan, highlighting cultural differences toward EOL care.^46^ This implies that healthcare providers in Taiwan and similar regions must consider culturally sensitive approaches to improve the uptake of palliative care services among older adults. Policymakers should prioritize educational programs that raise awareness on the benefits of palliative care and foster open discussions about EOL preferences within families to ensure that older individuals receive appropriate and timely care.

Significant gender disparities exist in EOL care, as shown by previous studies and our findings in Taiwan, which indicate that women are more likely than men to utilize palliative care services.^47^, ^48^, ^49^, ^50^ This difference could be influenced by living situations, symptom experiences, care contexts and preferences, caregiving roles, and coping strategies between genders.^47^ Women are traditionally socialized to be caring, sensitive, and self-sacrificing, often prioritizing others' needs over their own, even at personal cost.^51^ These deeply rooted beliefs significantly influence individuals’ perceptions of EOL expectations and shape the types of support, treatment, and communication from healthcare providers.^47^^,^^51^ Nevertheless, it remains unclear whether the observed sex differences in palliative care utilization reflect a fundamental difference in EOL care preferences between genders, or if they are driven by gender-specific decision-making influenced by caregiver preferences, marital status, and financial circumstances. Further research is required to explore how these factors impact EOL preferences/decision-making.

Income level significantly affects palliative care utilization, with disparities observed among different socioeconomic groups.^52^^,^^53^ However, in our study, we did not find a significant effect of insurance premiums on palliative care utilization after adjusting for covariates. A possible explanation is that Taiwan’s NHI covers the costs of palliative care and medication, particularly waiving out-of-pocket payments for low-income patients and families. This policy minimizes financial barriers that might otherwise prevent low-income individuals from pursuing palliative care. Future research should explore the long-term impact of financial support policies on palliative care access and utilization among low-income groups, examining whether these measures effectively address socioeconomic disparities in EOL care.

This study had the following limitations: First, claims data was used which may not capture all the factors influencing palliative care utilization, such as patients' preferences or family dynamics. Second, reliance on administrative data and ICD codes may have introduced potential exposure misclassification, particularly for non-cancer conditions, where coding practices can vary between physicians. However, we implemented several measures to ensure the accuracy and reliability of the data, including the use of well-defined inclusion criteria and validated ICD codes specifically linked to palliative care services. This careful selection process helped minimize the risk of misclassification by excluding ambiguous cases that did not clearly indicate palliative care. Additionally, we utilized the Multiple Causes of Death database to capture non-cancer conditions, which has been confirmed as valid. Third, the findings from Taiwan may not be directly applicable to other regions with different healthcare systems, cultural contexts, and palliative care policies, thus limiting the study’s generalizability. Fourth, we were unable to consider all potential confounders in our analysis mainly due to certain variables that were not measured in the NHI claims. This may have resulted in residual confounding. For example, patients with lower health literacy levels are often diagnosed with cancer at more advanced stages and have higher mortality rates.^54^ Health literacy enables patients and families to better understand palliative care, including its benefits and goals. This knowledge increases the likelihood of utilizing palliative care services when appropriate.^55^ Individuals with low health literacy may have limited knowledge of palliative care, leading to underutilization. Failure to consider health literacy may lead to increased differences in palliative care utilization between cancer and non-cancer decedents. Finally, the exclusion of individuals with missing demographic data or hospital characteristics could have caused a potential selection bias because these missing data points may be systematically different.

Despite these limitations, this study had several strengths. First, the study included a large number of deceased individuals (588,010) by using a population-based approach, which provided robust statistical power to identify trends in palliative care utilization and associations with various causes of death. Additionally, the population-based design offered representative information on palliative care utilization across Taiwan, reflecting real-world practices in a universal healthcare system. Second, the use of data spanning a decade allowed for the observation of utilization trends over time, highlighting changes in palliative care practices and policies. Third, we addressed a significant gap in the literature by examining the history of palliative care utilization among deceased non-cancer patients, a growing but often overlooked patient population. Our findings presented valuable information for policymakers aiming to improve palliative care access and equity, particularly for non-cancer patients and older adults living in less-urbanized areas.

### Conclusion

This study highlighted the significant increase in palliative care utilization among older adults who died in Taiwan from 2010 to 2020. However, disparities persist, with a notably lower proportion among non-cancer decedents. These findings emphasize the importance of overcoming cultural, systemic, and educational barriers. Policymakers and healthcare providers should focus on expanding services, improving awareness among patients/families, training physicians, and fostering cultural sensitivity to address the needs of an aging population. Taiwan’s approach serves as a model for integrating palliative care with standard healthcare practices.

## Contributors

YTL and YTH conceptualized this study. YLW and TJC collected the data. TJC had access to raw data and performed the analyses. CYL and YTH verified the data and provided feedback on the data analyses and interpretation. YTL contributed primarily to the first draft of the manuscript. CYL, YCY, YTH, and YLW provided feedback on the draft protocol and manuscript. CYL had final responsibility for the decision to submit for publication. All authors read and approved the final manuscript before its submission, agreed to its submission to the journal, and take responsibility for the content of the article.

## Data sharing statement

The data and resources were shared with eligible researchers through established academic channels. The data sets used in this study are available upon request from the corresponding author.

## Editor note

The Lancet Group takes a neutral position with respect to territorial claims in published maps and institutional affiliations.

## Declaration of interests

The authors declare that they have no known competing financial interests or personal relationships that could have influenced the work reported in this article.

## Appendix ASupplementary data

Supplementary Table S3

Translated abstract
