# Supplementary material for: Trends in palliative care utilization among older adult decedents with and without cancer in Taiwan: a population-based comparative study
Source: Lancet Reg Health West Pac. 2025 Jan 28;55:101479. doi: 10.1016/j.lanwpc.2025.101479 (PMC11814702; doi:10.1016/j.lanwpc.2025.101479)
Supplement: Supplementary Fig. S1 [file mmc1.docx]

Excluded:

<65 y/o at death, n = 525,822

Underlying cause of death irrelevant to palliative care, n = 629,997

Without records of inpatient or emergency utilization 6 months before death, n = 39,268

Missing demographic data such as sex, insurance premium, and region of residence, n = 2,215

Missing hospital level characteristics data, n = 11,685

Study population: n = 588,010

Patients died between January 1, 2010 and December 31, 2020, N = 1,796,997

**Supplementary Figure 1. Flow diagram of participant inclusion**
