# Supplementary material for: Trends in palliative care utilization among older adult decedents with and without cancer in Taiwan: a population-based comparative study
Source: Lancet Reg Health West Pac. 2025 Jan 28;55:101479. doi: 10.1016/j.lanwpc.2025.101479 (PMC11814702; doi:10.1016/j.lanwpc.2025.101479)
Supplement: Supplementary Fig. S2 [file mmc2.docx]

**
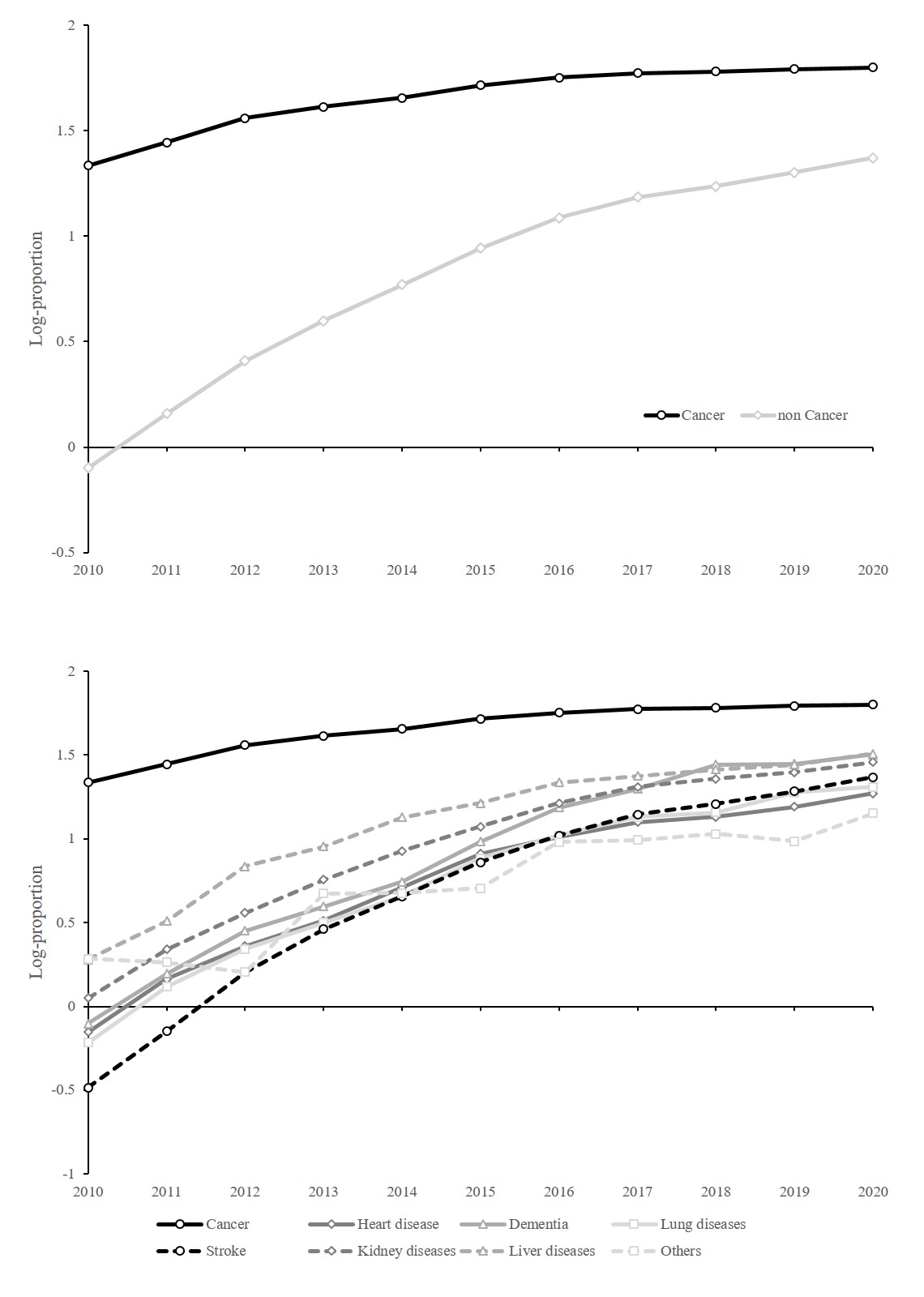
**

**Supplementary Figure 2. Scatter plot of the trend against the log-transformed proportion of palliative care utilization**
