# Supplementary material for: Trends in palliative care utilization among older adult decedents with and without cancer in Taiwan: a population-based comparative study
Source: Lancet Reg Health West Pac. 2025 Jan 28;55:101479. doi: 10.1016/j.lanwpc.2025.101479 (PMC11814702; doi:10.1016/j.lanwpc.2025.101479)
Supplement: Supplementary Table S2 [file mmc5.docx]

**Supplementary Table 2. List of ICD-9 and ICD-10 diagnostic codes for hospice palliative care**

| Diagnostic cluster | ICD-9 | ICD-10 |
| --- | --- | --- |
| Cancer | 140.x-208.x, 238.7, 273.3, 277.8 | C00.x-C97.x |
| Liver disease | 070.22, 070.23, 070.32, 070.33, 070.44, 070.54, 070.59, 456.0, 456.1, 456.8, 570, 571.x-573.x | B18.x, I85.0x, I86.4, K70.x, K71.0, K71.1x, K71.3, K71.4, K71.5x, K71.6, K71.7, K71.8, K71.9, K72.1x, K72.9x, K73.x, K74.x, K75.x, K76.x, K77 |
| Lung diseases | 415.19, 416.8, 416.9, 490.x–505.x, 506.4, 508.1, 508.8, 519.1  277.00 | I27.8x, I27.9, J40.x–J47.x, J60.x–J67.x, J68.4, J70.1, J70.2, J70.3  E84.0 |
| Stroke and others | 430.x–438.x, 340, 332.x, 333.0, 333.1 | G45.x, G46.x, H34.0, I60.x–I69.x, G35, G20, G21.x, G23.x, G10 |
| Kidney disease | 583.6, 583.7, 584.5, 584.6, 584.7, 584.8, 584.9, 585, 586, 593.9 | N17.x, N18.4, N18.5, N18.6, N18.9, N19, N18.0, N18.8 |
| Heart disease | 398.90, 398.91, 402.01, 402.11, 402.91, 404.01, 404.03, 404.11, 404.13, 404.91, 404.93, 414.8, 425.2, 425.4, 425.5, 425.7, 425.8, 425.9, 428.x, 779.8 | I09.81, I09.9, I11.0, I13.0, I13.2, I25.5, I42.0, I42.5–I42.9, I43.x, I50.x, P29.0 |
| Dementia | 290.x, 291.1, 291.2, 292.82, 294.1, 294.8, 294.9, 331.0, 331.1, 331.2 | F01.x-F03.x, F10.27, F10.97, F13.27, F13.97, F18.27, F18.97, F19.27, F19.97, G30.x, G31.x |
| Others  (Motor neuron diseases, Myelodysplastic syndromes, Age-related frailty,  Others,  Rare Disease) | 335.20, 335.21, 335.22, 335.24, 335.29  284.9, 285, 238.7  797  277.00, 331.7, 333, 333.4, 334.x, 335.10, 335.20, 335.21, 335.9, 349.89, 356.0, 359.0, 359.1, 416.0, 719.60, 719.61, 719.62, 719.63, 719.64, 723.5, 754.89, 757.39, 780.03, 781.3, 781.91, 781.99  237.72, 251.1, 253.2, 253.3, 255.1, 255.4, 255.5, 258.1, 259.4, 259.8, 266.2, 269.3, 270.0-270.8, 271.0, 271.1, 271.4, 271.8, 272.0, 272.3, 272.6-272.9, 275.1, 275.3, 275.49, 277.00, 277.1-277.3, 277.5, 277.6, 277.8, 277.9, 279.04, 279.05, 279.11, 279.12, 279.2, 279.3, 279.8, 282.4, 283.11, 283.2, 287.1, 287.8, 288.1, 289.8, 299.8x, 319, 330.0, 330.1, 330.8, 331.89, 333.0, 333.4, 333.91, 334.0., 334.1, 334.3, 334.8, 335.10, 335.20-335.22, 335.24, 335.29, 335.9, 340, 341.8, 345.11, 345.2, 345.3, 356.0-356.2, 359.2, 359.8, 362.70, 416.0, 448.0, 576.2, 628.1, 705.0, 728.11, 731.0, 742.2, 743.41, 743.44, 747.89, 751.5, 751.60, 751.69, 753.14, 755.38, 755.39, 755.53-755.59, 755.67, 755.69, 755.8, 756.0, 756.3, 756.4, 756.51, 756.52, 756.54, 756.54, 756.56, 756.59, 756.8x, 756.9, 757.1, 757.2, 757.31, 757.33, 757.39, 758.3, 758.8x, 759.1, 759.5-759.7, 759.81, 759.89, 775.1, 780.51, 780.53, 780.57, 785.59, 271.3+270.1 | G12.2x  D46.x  R54  E84.9, G10, G11.x, G12.21, G12.9, G31.2, G32.8x, G60.2, G71.0, G71.2, G90.3, I27.0, Q74.3, Q81.x, R27.x, R29.8xx, R40.3  D56.0, D56.1, D59.3, D59.5, D68.59, D71, D80.5, D81.0, D81.1, D81.2, D81.810, D81.819, D81.9, D82.0, D82.1, D82.4, D84.1, D84.8, E16.1, E20.1, E23.0, E26.81, E27.49, E31.0, E34.3, E34.8, E61.5, E70.0, E70.1, E70.20, E70.21, E70.41, E70.8, E70.9, E71.0, E71.110, E71.118, E71.120, E71.19, E71.3x, E71.41, E71.510, E71.511, E71.520, E71.521, E71.528, E71.529, E71.540, E72.03, E72.04, E72.11, E72.19, E72.20, E72.23, E72.29, E72.3, E72.4, E72.51, E72.52, E72.59, E72.8, E74.01, E74.02, E74.03, E74.04, E74.09, E74.21, E74.4, E74.8, E75.00, E75.11, E75.19, E75.2x, E75.4, E75.5, E76.x, E76.3, E77.0, E77.1, E77.8, E78.0, E78.3, E78.70, E78.71, E78.72, E79.1, E80.2x, E83.01, E83.09, E83.31, E83.32, E83.39, E84.9, E85.1, E88.01, E88.1, E88.40, E88.41, E88.49, E88.9, F78, F84.2, F84.8, G10, G11.1, G11.3, G11.4, G12.2x, G12.9, G23.0, G25.82, G31.82, G31.89, G35, G37.8, G40.311, G47.35, G60.0, G71.0, G71.11, G71.13, G71.2, G71.8, G87.0, H35.50, H49.811, H35.50, H49.81x, I27.0, I78.0, K83.1, L74.4, M61.10, M61.11x, M61.12x, M61.13x, M61.14x, M61.15x, M61.16x, M61.17x, M61.18, M61.19, M88.x, P70.2, Q04.3, Q13.4, Q28.8, Q43.8, Q44.7, Q61.19, Q71.6x, Q72.7x, Q74.0, Q74.8, Q75.1, Q75.4, Q77.2, Q77.3, Q77.4, Q77.8, Q78.0, Q78.1, Q78.2, Q78.3, Q79.6, Q79.8, Q80.2, Q80.3, Q80.4, Q81.9, Q82.3, Q82.4, Q82.8, Q85.02, Q85.1, Q85.8, Q87.0, Q87.1, Q87.2, Q87.3, Q87.89, Q89.1, Q89.7, Q89.8, Q93.5, Q93.88, Q93.89, Q97.8, Q98.8, Q99.8, E74.31+E70.0, E71.120+E72.11 |
