# Supplementary material for: Trends in palliative care utilization among older adult decedents with and without cancer in Taiwan: a population-based comparative study
Source: Lancet Reg Health West Pac. 2025 Jan 28;55:101479. doi: 10.1016/j.lanwpc.2025.101479 (PMC11814702; doi:10.1016/j.lanwpc.2025.101479)
Supplement: Supplementary Table S3 [file mmc6.docx]

**Supplementary Table 3. Diagnosis and other factors associated with palliative care utilization, treating age and calendar year as continuous variables.**

| **Factors** | Adjusted OR Model 2  (95% CI) | P value | Adjusted OR Model 3  (95% CI) | P value |
| --- | --- | --- | --- | --- |
| **Age (per 5 years)** | 0.97 (0.97 – 0.98) | <.0001 | 0.98 (0.98 – 0.98) | <.0001 |
| **Sex (Reference: Male)** |  |  |  |  |
| Female | 1.20 (1.19 – 1.22) | <.0001 | 1.20 (1.18 – 1.21) | <.0001 |
| **Diagnosis**  **(Reference: cancer)** |  |  |  |  |
| Non-cancer | 0.13 (0.13 – 0.13) | <.0001 | - |  |
| heart disease | - |  | 0.10 (0.10 – 0.10) | <.0001 |
| dementia | - |  | 0.20 (0.19 – 0.21) | <.0001 |
| lung diseases | - |  | 0.12 (0.12 – 0.12) | <.0001 |
| stroke | - |  | 0.11 (0.11 – 0.11) | <.0001 |
| Kidney diseases | - |  | 0.16 (0.16 – 0.17) | <.0001 |
| Liver diseases | - |  | 0.20 (0.19 – 0.21) | <.0001 |
| others | - |  | 0.08 (0.07 – 0.08) | <.0001 |
| **Year of death** | 1.22 (1.22 – 1.22) | <.0001 | 1.22 (1.22 – 1.22) | <.0001 |
| **Insurance premium**  **(Reference: ≤25000)** |  |  |  |  |
| >25000 | 1.00 (0.99 – 1.02) | 0.71 | 1.00 (0.99 – 1.02) | 0.6909 |
| **Level of urbanization** |  |  |  |  |
| **(Reference: low)** |  |  |  |  |
| Medium | 0.91 (0.89 – 0.92) | <.0001 | 0.91 (0.89 – 0.92) | <.0001 |
| High | 1.02 (1.01 – 1.04) | 0.0037 | 1.02 (1.01 – 1.04) | 0.0079 |
| **Hospital level**  **(Reference: Medical center)** |  |  |  |  |
| Regional hospital | 0.70 (0.69 – 0.71) | <.0001 | 0.70 (0.69 – 0.71) | <.0001 |
| District hospital | 0.26 (0.25 – 0.26) | <.0001 | 0.26 (0.25 – 0.26) | <.0001 |
