# Supplementary material for: Trends in palliative care utilization among older adult decedents with and without cancer in Taiwan: a population-based comparative study
Source: Lancet Reg Health West Pac. 2025 Jan 28;55:101479. doi: 10.1016/j.lanwpc.2025.101479 (PMC11814702; doi:10.1016/j.lanwpc.2025.101479)
Supplement: Translated abstract [file mmc7.docx]

**Editor Disclaimer:** This translation in Chinese was submitted by the authors and we reproduce it as supplied. It has not been peer reviewed. Our editorial processes have only been applied to the original abstract in English, which should serve as reference for this manuscript.

**Translated abstract**

**背景**

高齡者的死亡主因已從癌症轉變為心臟病、中風，與失智症等非癌症疾病，將直接影響臨終照護的需求。本研究旨在探討台灣於 2010 年至 2020 年間，不同死因對安寧療護利用率的影響，並比較癌症及非癌症高齡死亡個案間安寧療護利用率趨勢之差異。

**方法**

本研究利用台灣衛生福利部資料科學中心之健保資料，涵蓋 2010 年至 2020 年間 588,010 位 65 歲以上死亡個案之人口學及醫療利用變項。採用 Poisson 回歸分析探討生命最後六個月安寧療護利用率之時間趨勢變化。並且建構多變量邏輯斯迴歸模型，分析癌症及非癌症死因與安寧療護利用率之關聯性。

**結果**

癌症死亡個案之安寧療護利用率自 2010 年的 21.7% 提升至 2020 年的 63.2%，beta係數為 0.09 (95% CI: 0.09-0.09)。非癌症死亡個案之安寧療護利用率自 2010 年的 0.8% 提升至 2020 年的 23.5%，beta係數為 0.26 (95% CI: 0.26-0.26)。相較於癌症死亡個案，非癌症死亡個案接受安寧療護之可能性較低 (OR= 0.12, 95% CI: 0.12-0.13)。

**結論**

為確保非癌症個案公平使用安寧療護，未來應努力聚焦於擴大服務、提升照護者教育，以及促進文化敏感度，以滿足超高齡社會日益增長的安寧療護需求。
